# DNA sequence-induced solid phase transition as a solution to the genome folding paradox

**DOI:** 10.21203/rs.3.rs-5448201/v1

**Published:** 2024-12-05

**Authors:** Joan M. Pulupa, Natalie G. McArthur, Olga Stathi, Miao Wang, Marianna Zazhytska, Isabella D. Pirozzolo, Ahana Nayar, Lawrence Shapiro, Stavros Lomvardas

**Affiliations:** 1 Department of Biochemistry and Molecular Biophysics, Vagelos College of Physicians and Surgeons, New York, NY 10032, USA; 2 Mortimer B. Zuckerman Mind, Brain, and Behavior Institute, Columbia University, New York, NY 10027, USA; 3 Department of Biological Sciences, Columbia University, New York, NY 10027, USA; 4 Medical Scientist Training Program, Vagelos College of Physicians and Surgeons, New York, NY 10032, USA; 5 Barnard College, New York, NY, 10025, USA

## Abstract

Ultra long-range genomic contacts, which emerge as prominent components of genome architecture, constitute a biochemical paradox. This is because regulatory DNA elements make selective and stable contacts with DNA sequences located megabases away, instead of interacting with proximal sequences occupied by the same exact transcription factors (TF). This is exemplified in olfactory sensory neurons (OSNs), where only a fraction of Lhx2/Ebf1/Ldb1-bound sites interact with each other, converging into highly selective multi-chromosomal enhancer hubs. *In vitro hub* reconstitution reveals that TF motif variations impose distinct homotypic properties to their resident Lhx2/Ebf1/Ldb1 complexes, enabling formation of nucleoprotein condensates with solid phase characteristics. Live imaging and single molecule tracking of Lhx2/Ebf1 proteins in cultured OSNs confirm that assembly of transcription-competent solid condensates occurs *in vivo* under physiological protein concentrations. Thus, DNA sequence-induced homophilic nucleoprotein interactions provide a generalizable explanation for the stability and specificity of long-range genomic contacts that control cellular identity and function.

Tissue specific transcriptional activation requires genome folding reorganization that places distant enhancers near the promoters they regulate^1,2^. Intergenic enhancers usually activate promoters from the same topologically associated domain (TAD)^3^, however, inter-TAD enhancer-promoter contacts are both frequent and important for gene regulation^4–7^. From the original report on “transvection”^8^, to the discovery that limb expression of Sonic Hedgehog requires an intronic enhancer located ~850Kb away^9^, there is a plethora of examples of gene regulation over vast genomic distances in the chromosome^10^, or even across chromosomes^11–20^. Inter-TAD or inter-chromosomal genomic interactions, however, present a biochemical paradox, for two main reasons: First, transcription factors (TFs) have short residence time on the DNA^21,22^, therefore TF-induced DNA loops should be highly dynamic, as shown for cohesin/CTCF-dependent DNA contacts^23,24^. While dynamic regulatory interactions are compatible with transcription^25,26^, ultra long-range interactions assemble over a period of several days^4^, thus cannot afford to fall apart and reform at the rapid rate of DNA loops. Second, the same TFs that orchestrate ultra long-range contacts between two DNA elements also occupy thousands of additional genomic sites that are excluded from these interactions, raising important questions about the source of specificity in genome architecture. To obtain insight to the principles of genome folding stability and specificity, we turned to olfactory sensory neurons (OSNs), which generate extensive networks of highly specific, ultra long-range *cis* and *trans* enhancer contacts to establish gene expression programs essential for odor detection.

OSNs have an “inverted” nuclear architecture characterized by a centrally located heterochromatic core^27–29^, and extensive long-range *cis* and *trans* genomic interactions^4,7,29–31^, variations of which were recently detected in many other neuronal populations^32,33^. This intricate network of highly specific and stable genomic contacts likely contributes to every facet of OSN identity and function, as it regulates both the monogenic and monoallelic olfactory receptor (OR) choice^34^, as well as, gene expression programs involved in OR processing and OR signaling^35^. Key components of this regulatory paradigm include the LIM-homeodomain TF Lhx2 and the LIM domain binding protein Ldb1^4,36^. In the case of OR gene regulation, Lhx2/Ldb1 binding on the intergenic OR enhancers, the Greek Islands(GIs)^7,31,36,37^, facilitates assembly of Greek Island Hubs (GIHs), which support strong and, eventually, singular OR transcription for the life of the OSN^4,7,29–31^. The same two proteins bind on thousands of putative intergenic enhancers across the OSN genome, regulating a plethora of OSN-specific gene expression programs^4,27,35,36^. Yet, while there are >10,000 Lhx2/Ldb1 co-bound sites in the OSN genome, only a fraction participate in long range genome interaction networks. This selectivity is even more striking in the case of GIHs, which may contain GIs that are 80Mb apart in the chromosome^4,30^, and even GIs from other chromosomes^4,7,30,31^, but exclude non-GI sequences occupied by the same exact proteins. TFs Ebf1–4, which bind to every GI^36^, also occupy a large fraction of the Lhx2/Ldb1 peaks genome wide^36^, offering marginal improvement to our understanding of the source of specificity in these long range genomic interactions. In fact, the only genetic feature that distinguishes GIs from other putative intergenic enhancers bound by the same proteins, is the strong enrichment for the “composite” motif, an essential OR gene expression motif that consists of Lhx2 and Ebf binding sites stereotypically separated by 1bp^36^. We hypothesized that the composite motif, together with other sequence features of the Greek Islands, instruct the stability and specificity of GIH assembly. Thus, to obtain insight on the biochemical mechanisms by which DNA sequences orchestrate ultra long-range interactions, we first sought to reconstitute GIHs *in vitro,* using recombinant Lhx2/Ebf/Ldb1 proteins.

## Lhx2/Ebf1/Ldb1 and Greek Islands assemble large nucleoprotein complexes *in vitro*

**Extended Data Fig 1:**
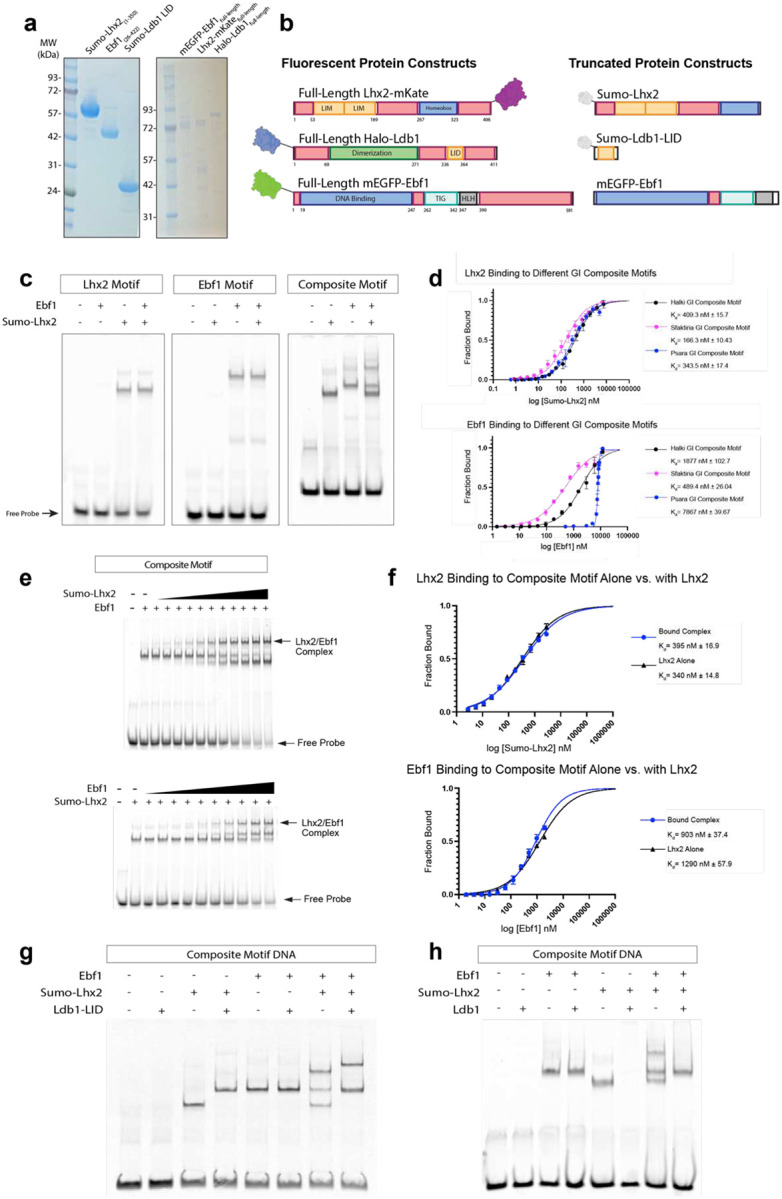
The proteins of the OR Hub co-bind to the composite motif. **a,** Coomassie stained SDS-PAGE gel of recombinant truncated and full-length Lhx2, Ebf1, and Ldb1 proteins. **b,** Schematic of the recombinant truncated and full-length proteins purified. **c,** Electromobility shift assay (EMSA) of reactions containing truncated Lhx2 and Ebf1 with Lhx2 motif, Ebf1 motif, and composite motif DNA. **d,** EMSA Binding curves and K_d_ values from reactions containing truncated Sumo-Lhx2 (top) and truncated Ebf1 (bottom) with three different composite motifs from Halki, Sfaktiria, and Psara Enhancers. **e,** Example EMSA from reactions quantified in f. **f,** Binding curves and K_d_ values from reactions containing truncated composite motif DNA with Lhx2 alone and in complex with Ebf1 (top) and with Ebf1 alone and with Lhx2 (bottom). **g,** EMSA of reaction containing truncated Sumo-Lhx2, Ebf1, and Sumo-Ldb1-LID domain with composite motif DNA. **h,** EMSA of reaction containing truncated Sumo-Lhx2, Ebf1 and full-length Ldb1 with composite motif.

We first expressed and purified truncated recombinant Lhx2 and Ebf1 proteins that lack C-terminal Intrinsically Disordered Regions (IDRs) (Extended Data Fig.1a, b). Electrophoretic mobility shift assays (EMSAs) confirm that the two TFs are properly folded and exhibit DNA binding specificity, as they bind on their respective consensus motifs, but not to each other’s motifs (Extended Data Fig. 1c). The two TFs also bind on the composite motif individually and simultaneously, suggesting that there is no steric hindrance between Lhx2 and Ebf1 proteins when their respective motifs are 1bp apart (Extended Data Fig. 1c). Similar observations were made when we compared composites from various GIs by EMSA, which revealed that Ebf1 has variable affinity for composites that is 5–20fold lower than the affinity that Lhx2 has for the same motifs (Extended Data Fig.1d). However, while the two proteins co-bind on the composite motif, they do not bind cooperatively (Extended Data Fig. 1e, f). To ask if the two TFs synergize for the recruitment of Ldb1, we first expressed and purified a truncated Ldb1 protein that contains the LIM interaction domain (LID) but lacks dimerization domains^38,39^ (Extended Data Fig. 1a, b). Addition of the truncated Ldb1 protein produced a migration shift consistent with Ldb1 recruitment by Lhx2 and Lhx2/Ebf1 complexes, but not by Ebf1 alone (Extended Data Fig. 1g). To test if Ebf1 interacts with other regions of the Ldb1 protein we repeated the EMSA using full length Ldb1. Unexpectedly, addition of full length Ldb1 protein to the binding reaction eliminates the shifts corresponding exclusively to the Lhx2-containing complexes (Lhx2 alone and Lhx2/Ebf1 co-bound) without affecting the Ebf1/DNA complex (Extended Data Fig. 1h). These observations show that Ldb1 is recruited to the composite motifs predominantly via direct interactions with the LIM domains^40^ of Lhx2 and suggest that the full length Ldb1 protein generates high molecular weight complexes that cannot migrate on EMSA gels. Alternatively, Ldb1 could induce phase separation of Lhx2-containing complexes, preventing migration to the gel. Either one of these two explanations, which are not mutually exclusive, may represent biochemical features evolutionarily selected for the regulation of long-range genomic interactions^41^, an extreme manifestation of which is found at GIHs.

**Extended Data Fig 2:**
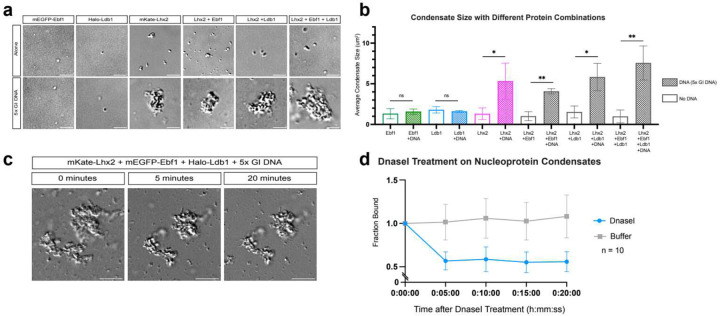
Characterization of the solid-like condensates formed by Lhx2, Ebf1, Ldb1, and Greek Island DNA *in vitro*. **a,** Representative DIC images of condensates formed in various combinations of mEGFP-Ebf1, Halo-Ldb1, and mKate-Lhx2, with and without 5x GI DNA. **b,** DIC quantification of the average condensate size formed in the conditions imaged in a (error bars show standard deviation across replicates, significance using unpaired t test) **c,** Representative DIC image of a condensate formed by mKate-Lhx2, mEGFP-Ebf1, Halo-Ldb1, and 5x GI DNA before DnaseI digestion (left), after 5 minutes of DnaseI digestion (middle), and after 10 minutes of DnaseI digestion (right) **d,** DIC quantification of the average condensate size after 10 minutes of DnaseI digestion in comparison to buffer control (error bars show standard deviation across replicates, n = 10). Scale bar for all images is 5 μm.

**Fig 1:**
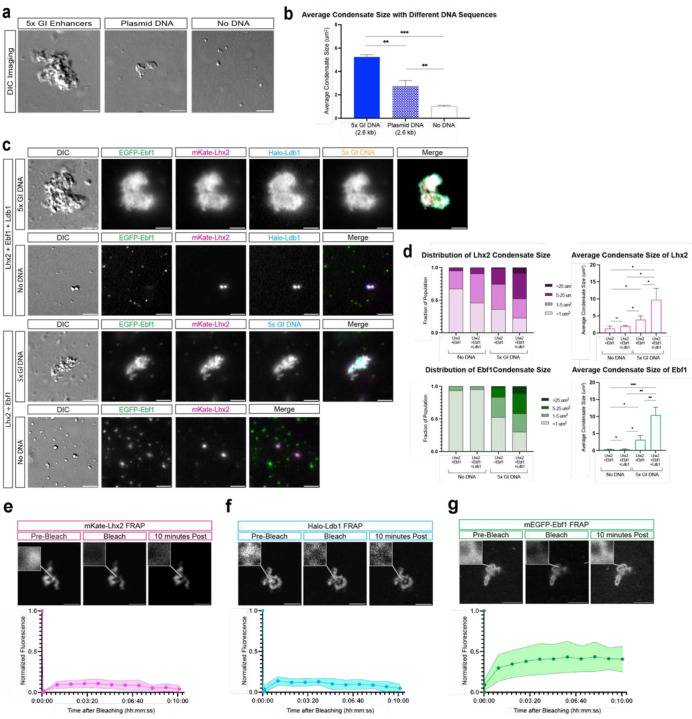
Nucleoprotein condensates with solid phase properties assemble over GI DNA *in vitro*. **a,** Representative DIC Imaging of reactions containing mEGFP-Ebf1, mKate-Lhx2, and Halo-Ldb1 with 5x- GI DNA, linear plasmid DNA, and no DNA. **b**, DIC quantification of the average condensate size formed by Lhx2, Ebf1, and Ldb1 in the three different reactions imaged in a (error bars show standard deviation across replicates, significance using unpaired t test). **c,** Representative images of reactions containing combinations of mEGFP-Ebf1, mKate-Lhx2, and Halo-Ldb1 with and without 5x GI DNA. **d,** Fluorescence quantification of Lhx2 and Ebf1 measuring the distribution of Lhx2 and Ebf1 condensate size with and without DNA added. Condensate size is grouped and colored by size groupings of <1um^2^, 1–5 μm^2^, 5–25 μm^2^ and >25 μm^2^ (left). Fluorescence quantification of the average condensate size formed by Lhx2 and Ebf1 fluorescence channels (error bars show standard deviation across 3 replicates, significance using unpaired t test) (right). **e-g,** Fluorescence recovery after photobleaching (FRAP) on condensates containing mKate-Lhx2, mEGFP-Ebf1, Halo-Ldb1, and enhancer DNA. FRAP of mKate-Lhx2 (left), Halo-Ldb1 (middle), and mEGFP-Ebf1 (right). Recovery is plotted over a timescale of 10 minutes and represents the averages of 4 replicates. Scale bar for all images is 5 μm.

To ask if Lhx2/Ebf1/Ldb1 complexes bound GIs can form GIHs *in vitro*, we expressed full length proteins (Extended Data Fig.1a, b), which we incubated with a 2.6Kb DNA template that contains 5 different GI enhancer sequences^36^, mimicking the nucleoprotein constitution of a GIH. Strikingly, within minutes Lhx2/Ebf1/Ldb1 and Greek Island DNA form large complexes visible by differential interference contrast (DIC) microscopy (Fig. 1a, b). Incubation of the same proteins with a linear plasmid DNA of the same size, or without any DNA, results in formation of puncta that are significantly smaller than the complexes formed in the presence GI DNA (Fig. 1a, b). Incubation of the GI DNA template and various protein combinations revealed a seeding role of Lhx2 in this process, which is further enhanced by the addition of Ldb1 and Ebf1 proteins (Extended data Fig. 2a, b). Specifically, the largest nucleoprotein complexes form only in the presence of Lhx2/Ebf1/Ldb1, but Lhx2, Lhx2/Ebf1, and Lhx2/Ldb1 also form visible complexes in the presence of GI DNA (Extended data Fig. 2a, b). To further dissect the properties of these complexes, we fluorescently labeled the DNA template and used fluorescence microscopy to deduce the distribution of full-length Lhx2-mKate, mEGFP-Ebf1, and Halo-Ldb1 protein fusions (Extended Data Fig. 1a). Consistent with the DIC data, incubation of fluorescent Lhx2/Ebf1/Ldb1 proteins with GI DNA results in formation of large complexes that contain all three proteins and the labeled DNA (Fig. 1c, d). Considering that the GI DNA is end-labeled and not detectable in solution, strong DNA fluorescence in these complexes represents local concentration of multiple Lhx2/Ebf1/Ldb1-bound DNA molecules and, thus, successful reconstitution of GIHs *in vitro*. Without Greek Island DNA, the same three proteins form small puncta with the strongest fluorescence signal consisting of either Ebf1 or Lhx2/Ldb1 proteins, but rarely all three. Interestingly, as eluded by DIC microscopy, Lhx2- and Lhx2/Ebf1-GI DNA complexes can form without Ldb1, however, they are significantly smaller than Ldb1-containing complexes (Fig. 1c, d).

## Greek Island hubs have solid phase properties *in vitro*

DIC and fluorescent microscopy revealed that Lhx2/Ebf1/Ldb1/DNA nucleoprotein complexes are not round like liquid droplets formed by TF/co-activator/DNA complexes reported in other systems^42,43^. Moreover, while these complexes continue to grow under the microscope, we do not observe “fusion” of individual complexes as reported for liquid droplets^44^, suggesting that if these complexes represent condensates, they do not have liquid properties. Indeed, fluorescent recovery after photobleaching (FRAP) experiments showed no recovery of fluorescence for Lhx2 and Ldb1 even 10 minutes post photobleaching specific spots on the GI condensates (Fig.1e, f). While Ebf1 fluorescence partially recovers over time, this recovery is incomplete (40%) and significantly slower (3–5 minutes) (Fig. 1e) than the recovery observed for Ebf1 in liquid droplets, which is fully restored within 40 seconds^45^. To further interrogate the properties of the nucleoprotein condensates formed by GI DNA, we incubated already formed condensates with DNaseI and measured their average size over time.

Within 5 minutes, DNaseI treatment reduces the average condensate size by half, but additional digestion does not eliminate the remaining complex (Extended Data Fig.2c, d), suggesting that the core of these condensates is extremely stable either because the DNA is inaccessible or because the intermolecular interactions between Lhx2/Ebf1/Ldb1 complexes are too strong to dissolve. These observations, taken together, suggest that GI DNA acts as a scaffold that organizes Lhx2/Ebf1/Ldb1 complexes into large nucleoprotein condensates with solid phase properties, mimicking previously described functions of RNA molecules in the assembly of gel-like condensates^46,47^. We therefore asked if this unusual phase transition is caused by distinct sequence features of the GI DNA, which would account for the unique ability to form multi-chromosomal GIHs *in vivo*.

## DNA sequence influences the properties of Lhx2/Ldb1/Ebf1 nucleoprotein condensates

**Fig. 2:**
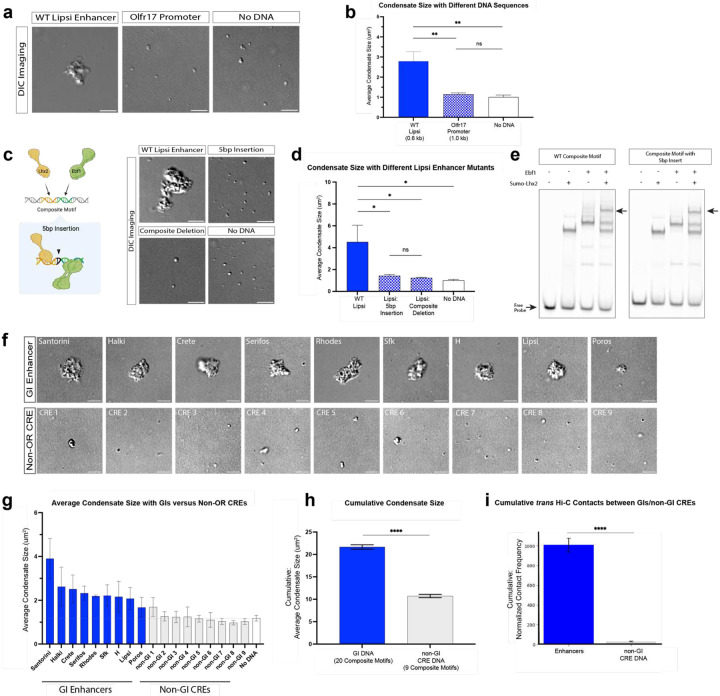
DNA sequence influences the ability of Lhx2/Ebf1/Ldb1 complexes to form nucleoprotein condensates. **a,** Representative DIC images of reactions containing mEGFP-Ebf1, mKate-Lhx2, and Halo-Ldb1 with Lipsi enhancer DNA, Olfr17 promoter DNA, and no DNA. **b,** DIC quantification of the average condensate size formed from the replicate image reactions shown in panel a (error bars show standard deviation across 3 replicates, significance using unpaired t test). **c,** Schematic of the 5bp insertion mutation to the composite motif (left). Representative DIC images of reactions containing mEGFP-Ebf1, mKate-Lhx2, and Halo-Ldb1 with wild type (WT) Lipsi enhancer DNA, Lipsi with the 5bp motif insertion mutation, Lipsi with the composite motif deletion mutation, and no DNA (right). **d,** DIC quantification of the average condensate size formed from the 3 replicate image reactions shown in panel c (error bars show standard deviation across replicates, significance using unpaired t test). **e,** EMSA using truncated Sumo-Lhx2 and Ebf1 protein with WT composite motif DNA and composite motif DNA with the 5bp insertion mutation. Arrows indicate free probe and the Lhx2/Ebf1 protein complex. **f,** Representative DIC images of reactions containing mKate-Lhx2, mEGFP-Ebf1, and Halo-Ldb1 with 9 different GI enhancers and 9 different non-GI CREs. **g,** DIC quantification of the average condensate size formed by the reactions in g (error bars show standard deviation across X replicates, significance using unpaired t test). **h,** Plot of the cumulative average condensate size formed by all enhancers and CREs from g. **i,** Quantification from *in vivo* Hi-C data showing the *trans* contact frequency made by the nine enhancers tested in h and the *trans* contact frequency made by the non-GI CREs tested in h. Scale bar for all images is 5 μm

To decipher the DNA sequence requirements for solid phase separation, we first asked if individual GIs, rather than GI arrays, can form nucleoprotein condensates. Indeed, a 600 bp DNA fragment containing the GI Lipsi forms large condensates, confirming that an individual GI contains sufficient genetic information for the seeding of intermolecular enhancer complexes with solid properties (Fig. 2a, b). In parallel, we performed the same assay using a 1Kb DNA fragment containing the promoter of OR gene Olfr17 (referred to as OR promoter DNA). Intriguingly, side by side comparison of the two sequences shows that Lipsi forms significantly larger condensates than the OR promoter (Fig. 2a, b), despite being 60% shorter. This observation is consistent with HiC and Dip-C experiments showing that Greek Islands participate in GIHs in significantly higher frequency than the OR promoters^4,30^, even though there are ~20 times more OR promoters than OR enhancers.

While both enhancer and promoter DNA fragments contain individual Lhx2 and Ebf motifs, composite motifs are significantly enriched on GIs and, reciprocally, significantly depleted from OR promoters^36^. Thus, we examined directly the role of the four composite motifs of Lipsi in the assembly of these condensates. We first deleted the four composites, resulting in significant reduction of the average size of nucleoprotein condensates (Fig. 2c, d). However, because composite deletions reduce the total number of Lhx2 and Ebf binding sites on Lipsi, we also generated composite mutations that do not alter the total number of Lhx2/Ebf1 motifs on this enhancer. Specifically, we inserted 5bp linkers between the Lhx2 and Ebf binding sites of the four composite motifs, leaving the Lhx2 and Ebf binding sites in each composite intact, while altering the relative protein orientation on the DNA helix^48^ (Fig. 2c). Strikingly, this insertion causes significant reduction in average condensate size, which is as strong and significant as the complete deletion of the four composites (Fig. 2c, d). Since EMSAs show that Lhx2 and Ebf1 binding on the composite motif is not affected by this 5bp insertion (Fig. 2e), we conclude that this insertion causes a significant structural rearrangement of the Lhx2/Ebf1/Ldb1 complex on the DNA that may prevent homotypic interactions with complexes formed on other Lipsi DNA molecules. Taken together, these observations strongly suggest that it is not just the number, but the exact sequence (or the exact arrangement) of Lhx2 and Ebf motifs that influence assembly of nucleoprotein condensates with solid phase properties *in vitro*, probably due to the generation of unique recruiting surfaces^49^ that promote strong interactions between nucleoprotein complexes in *trans*.

To further explore the role of DNA sequence in the assembly of nucleoprotein condensates, we amplified additional GI sequences and putative *cis* regulatory elements (CREs) that are bound by Lhx2/Ebf/Ldb1 in OSN nuclei but are not recruited to the GIHs. While there is variability in the condensate-promoting properties of the 18 DNA elements tested, GIs tend to form larger condensates than non-GI CREs, with 6/9 Greek Islands forming significantly larger condensates than the “no DNA” control, while 0/9 non-Greek Island CREs do (Fig. 2f, g). Moreover, if we compare the two types of sequences in aggregate, we find that GI condensates are significantly larger than the non-GI CRE condensates (Fig. 2h). This cumulative difference between the two types of DNA elements corelates with the difference in total numbers of composite motifs and the differences in ability to form interchromosomal contacts *in vivo* (Fig. 2i). Taken together, these results suggest that GIs, with the highest enrichment of composite motifs genome wide, utilize this sequence towards the assembly of multi-chromosomal hubs involved in OR gene regulation. However, given that composite motifs are also found in non-GI CREs, this sequence may be utilized by the rest of the genome for the assembly of enhancer interaction networks that regulate OSN-specific gene expression. Additional motif combinations and their relative arrangements may influence the homotypic properties of Lhx2 and/or Ebf1 proteins^50^, with the composite motif representing the first example of a TF binding sequence that distinctly promotes solid phase transitions of multi-enhancer complexes.

## Establishing an *in vivo* imaging system for TF solid phase transitions

**Extended Data Fig 3:**
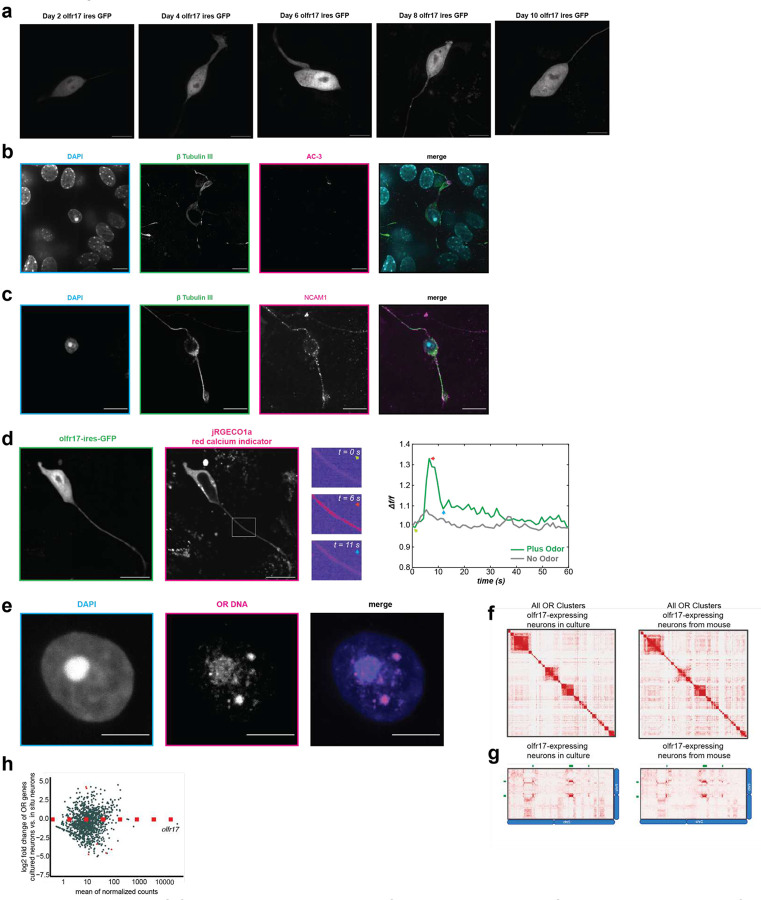
Olfr17-expressing Neurons in Culture Retain *in vivo* Characteristics. **a,** Live olfr17-ires-GFP neurons exhibit nuclear inversion, bipolar morphology, and GFP expression for at least 10 days in culture. Acquisition parameters and look up tables are consistent across all images. All image scale bars are 5 μm. **b,** Log2fold change of olfactory receptor mRNA between GFP-expressing cultured neurons 5 days in culture compared with GFP-sorted neurons from animals. **c-d**, Immunofluorescence of neurons in culture. Neurons express neuronal markers of mOSNs. **e,** Calcium Imaging of OSN in culture infected with jRGECO1a, a red calcium indicator. Cells were imaged every 1 second. At time 0, olfr17 ligand or vector alone were introduced. **f,** OR Loci form condensates in cultured neuron. DNA Fish with Pan-OR probe on cultured OSN. **g,** HiC contact maps between OR Clusters from cultured neurons (left) and neurons from animal (right) from pooled HiC data. Pixel intensity represents normalized number of contacts between pair of loci. **h,** HiC contact map between chromosome 2 (x-axis) and chromosome 9 (y-axis). Genomic position of OR clusters indicated as green bars.

To explore the physiological relevance of our *in vitro* observations we established a culturing protocol for OSNs that preserves their nuclear architecture and is compatible with live protein and DNA imaging. We used the recently described Gng8-tTA/tetO-Olfr17-iresGFP line, which stably expresses OR Olfr17 in the majority of OSNs^30^. An important feature of this mouse strain is that Olfr17 induction by the transiently expressed tTA in OSN progenitors, results in biased GIH assembly over the Olfr17 allele, and stable, tTA-independent expression in OSNs^30,51^. Dissociation of perinatal MOEs and culturing of the cells in the presence of fetal mouse astrocytes^52^, yields numerous GFP^+^ cells, which continue to express GFP for at least 10 days (Extended Data Fig.3a). These cells express neuronal (beta tubulin III) and OSN markers, including NCAM1 and Adcy3, which is properly localized at the cilia (Extended Data Fig.3b, c). Moreover, GFP^+^ cells respond to an Olfr17 ligand, Trans-2-undecenal (Dr. Sandeep Datta, personal communication), suggesting that key OR trafficking and signaling molecules are properly expressed for the duration of the culture (Extended Data Fig.3d). Importantly, the nuclei of the GFP^+^ cells have the characteristic inverted OSN organization *in vivo*, with a dense DAPI-positive chromocenter that corresponds to the centrally located OSN heterochromatin^27,28^. DNA FISH of the previously described pan-OR probe^27^, which labels most OR genes from the mouse genome, reveals genomic compartmentalization of OR sequences in the cultured OSNs, mimicking the developmentally dependent OR compartmentalization described in mature OSNs *in vivo*^4,27^ (Extended Data Fig.3e). Furthermore, *in situ* HiC of the GFP^+^ OSNs after 6 days in culture shows strong interchromosomal contacts between OR clusters, confirming that our culturing conditions do not disrupt the exquisite nuclear architecture previously described in primary OSNs (Extended Data Fig.3f,g)^4,30^. Finally, RNA-seq of the cultured GFP^+^ OSNs and direct comparison with primary GFP^+^ OSNs shows equally strong Olfr17 transcription, while there are no significant changes in the expression of the rest of the OR repertoire, confirming that OR expression remains monogenic and monoallelic after 6 days in culture (Extended Data Fig.3h).

**Extended Data Fig 4:**
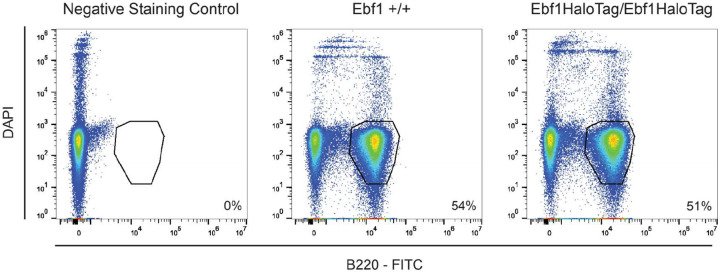
Ebf1-HaloTag Homozygotes Produce B Cells. Analysis of splenic B-cells analyzed by flow cytometry. B cells from the spleen of wildtype and Ebf1-HaloTag/Ebf1-HaloTag mice were stained with FITC-anti-B220 antibodies. 200k cells were analyzed per sample and percentages of cells for the indicated subpopulation is given. No antibody control (left), wildtype control (center), and Ebf1-HaloTag homozygotes (right) are shown.

**Fig 3:**
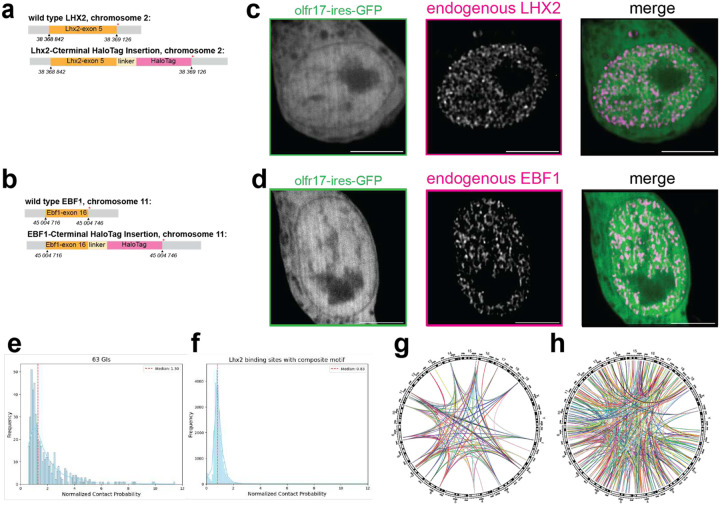
Visualizing endogenous Lhx2 and Ebf1 proteins in cultured OSNs. **a-b,** Schematics of Lhx2-HaloTag and Ebf1-HaloTag CRISPR insertions. **c-d,** Representative images of endogenous Lhx2-HaloTag protein or Ebf1-HaloTag protein labeled with JF646 HaloTag Ligand in live olfr17-expressing neurons. All scale bars are 5 μm. **e-f,** a, Trans contacts between 63 GIs b, Trans contacts between Lhx2 binding sites with composite motifs (excluding GIs). The red dashed line represents the median value of normalized contact probability. **g-h,** Circos plots show strong trans contacts within GIs, and trans contacts within Lhx2 bounding sites with composite motifs at cut off value of 2 for ICE-normalized contact probability (Threshold details outlined in e-f).

To image Lhx2 and Ebf1 protein dynamics, we introduced a HaloTag^53^ in the endogenous Lhx2 and Ebf1 alleles in fertilized mouse oocytes using CRISPR/Cas9, generating C-terminal HaloTag fusions for both proteins (schematic Fig.3a,b). We chose to generate knock-in mice for two reasons. First, we sought to preserve the endogenous Lhx2 and Ebf1 concentration in OSN nuclei, as TF levels are critical in the study of phase separation. Second, we can use the homozygote knock-in mice to confirm that HaloTag insertion does not disrupt Lhx2 or Ebf1 activity, because Lhx2 KO mice are embryonic lethal and Ebf1 KO mice lack B lymphocytes. Thus, homozygote Lhx2-HaloTag mice would have severe developmental defects, and homozygote Ebf1-HaloTag mice would have B cell differentiation deficiencies if HaloTag fusions disrupted Lhx2 and Ebf1 activity, respectively. However, homozygote Lhx2-HaloTag knock-in mice are viable, they are born in mendelian ratios, and they have fully developed MOEs, whereas homozygote Ebf1 mice have normal numbers of B lymphocytes (Extended Data Fig.4), suggesting that both HaloTag transcription factors are fully functional. We thus crossed Lhx2-HaloTag and Ebf1-HaloTag knock-in mice to Gng8-tTA/tetO-Olfr17iresGFP mice, and cultured primary OSNs from the triple transgenic strains, with the goal of determining the distribution and dynamics of Lhx2 and Ebf1 proteins in OSN nuclei. Intriguingly, we detected numerous Halo-labeled Lhx2 and Ebf1 foci representing local protein accumulation (Fig. 3c, d). If this focal TF distribution represents underlying multi-enhancer hubs, as in our *in vitro* experiments, then it would be consistent with the observation that every OSN contains ~6 major GIHs and 10s of additional smaller hubs representing pairs or triplets of interacting Greek Islands. Beyond GIHs, these foci may also represent multi-enhancer hubs assembled by non-Greek Island CREs that contain composite motifs bound by Lhx2/Ebf1/Ldb1 or different motif variations that may promote strong long-range interactions and solid phase transitions. Consistent with this, if we calculate the median strength of GI-GI *trans* contacts in Olfr17-expressing OSNs, we identify hundreds of *trans* interactions between Lhx2-bound, composite-containing CREs in the OSN genome, at 5Kb resolution (Fig. 3e–h). Although these non-GI *trans* contacts do not reach the strength of the most prominent GI-GI contacts in Olfr17-expressing OSNs, this result suggests that the composite sequence may drive assembly of large numbers of multi-enhancer hubs in OSNs, explaining our imaging data.

## Lhx2- and Ebf1- containing condensates have solid phase properties *in vivo*

**Fig 4:**
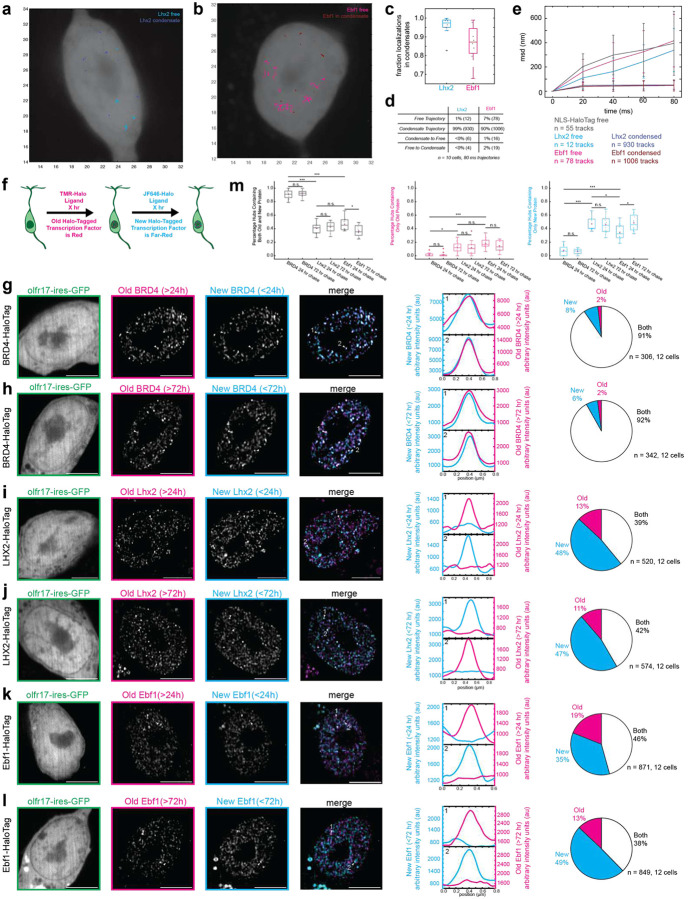
LHX2- and EBF1- Condensates Have Solid Phase Properties *in vivo*. **a-b,** Single molecule trajectories of (a) Lhx2-HaloTagged and (b) Ebf1-HaloTagged proteins labeled with JF646 HaloTag Ligand and imaged via bi-plane super resolution microscopy in a live cell. Light colored trajectories represent free trajectories and dark colored trajectories represent trajectories within a condensate. **c,** The fraction of protein localizations located within condensates. n = 10 cells. **d,** Distribution of trajectories (≥ 80 ms, 5 localization steps) into condensate or free. **e,** Mean squared displacement versus time of observed trajectories. Error bars represent standard deviation. **f,** Schematic of pulse chase experiment for Halo-Tag proteins in cultured OSNs. **g-l,** HaloTag fusion proteins in live OSNs differentially labeled with TMR HaloTag Ligand (old protein) and JF-646 HaloTag Ligand (new protein). Line scans are marked on figures with dotted white lines and arbitrary intensity units are plotted over distance. Pie charts show fraction of hubs containing only old protein (magenta), only new protein (cyan), and both old and new protein (white). OSNs are 5–6 days old. BRD4-HaloTag (**g-h**) is virally expressed. Lhx2-HaloTag (**i-j**) and EBF1-HaloTag (**k-l**) are endogenously expressed. All scale bars are 5 μm. **m,** Percentage of transcription factor hubs per cell containing both old and new protein (gray), only old protein (magenta), and only new protein (cyan). (n = 10 cells, boxes indicate quartiles, center bars indicate medians, red crosses represent outliers, magenta stars indicate means,* = p<0.05 , *** = p<0.0005, one-way ANOVA with post-hoc Tukey test)

To decipher Lhx2 and Ebf1 dynamics in the OSN nuclei, we performed single molecule tracking (SMT) with live imaging experiments in cultured OSNs. We compared the mobility of Lhx2 and Ebf1 in their respective foci, to the mobility of these protein molecules when they are singular in the nucleoplasm. We also infected OSNs with HaloTag containing a nuclear localization signal (HaloTag-NLS), which allows us to measure HaloTag mobility in the OSN nucleus in the absence of restrictive interactions with the DNA and protein partners. Although most Lhx2 and Ebf1 protein molecules in GFP^+^ OSNs are organized in discrete foci, the few unincorporated Lhx2 and Ebf1 protein molecules that we were able to track approach the mobility of HaloTag-NLS molecules, as they are displaced by an average of 350nm (Lhx2) and 400nm (Ebf1 and HaloTag-NLS) within 80ms. In contrast, at the same timeframe, Lhx2 and Ebf1 protein molecules within protein foci cover less than 10nm, providing strong evidence for solid phase properties of Lhx2- and Ebf1-containing condensates *in vivo* (Figure 4a–e).

To track Lhx2 and Ebf1 protein dynamics over longer time frames, we leveraged the fact that HaloTag proteins obtain their fluorescent properties upon covalently binding to their exogenously provided ligands, irreversibly labeling any HaloTag proteins expressed at a given moment. This property, together with the fact that different ligands can ascribe distinct excitation and emission properties to the HaloTag, enables a pulse/chase strategy for differential labelling of “old” and “new” proteins^54^. In this scheme, we first provide a “red” Halo ligand at saturating concentrations, labeling all the existing Lhx2 or Ebf1 proteins for 24 hours. Then, we wash away the unincorporated ligand, and add a second, “far-red” Halo ligand, at a window of 24 or 72 hours, which would label only proteins that were not previously labeled, i.e. proteins expressed after the first ligand was washed away (schematic of the experiment in Fig. 4f). To assure that this approach provides a sensitive measure for protein turnover in condensates, we infected OSNs with a HaloTag-Brd4 protein fusion. Brd4 constitutes one of the *bona fide* nuclear proteins with *in vivo* liquid separation properties^55^. Consistent with these liquid properties, we detect assembly of numerous Brd4 condensates in the OSN nucleus that are labeled with the red ligand, which was provided first. Moreover, we find that if we provide the far-red ligand 24- or 72-hours later, ~90% of the Brd4 condensates contain both new and old proteins (Fig. 4g, h), supporting rapid and continuous exchange of Brd4 protein molecules in liquid condensates, consistent with previously reported *in vivo* FRAP recovery in seconds^55^. In sharp contrast, Lhx2 and Ebf1 condensates predominantly contain proteins of the same age (i.e. only red or far-red proteins per condensate), with only 39% and 46% of the condensates containing both far-red and red-labeled Lhx2 and Ebf1 proteins, respectively (Fig. 4i, k). This statistically significant difference from the Brd4-containing liquid condensates is also observed at the 72-hour window (Fig. 4m). Thus, both at a sub-second and at a multi-day time scale, our live imaging experiments confirm that Lhx2 and Ebf1 have the propensity to undergo solid phase transitions in OSN nuclei, generating condensates with very slow protein turnover.

## Solid condensates physically engage with the transcriptionally active OR *in vivo*

**Fig 5:**
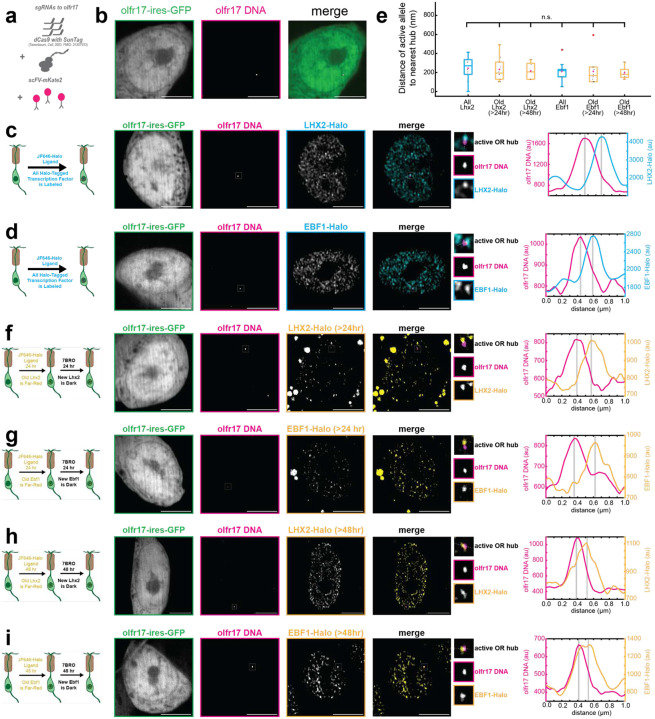
Stable LHX2 and EBF1 Condensates Localize to Active OR Allele *in vivo*. **a,** Schematic of OR allele labelling. **b,** olfr17 DNA visualized in olfr17-expressing, living OSN. **c-d,** Lhx2 and Ebf1 form hubs adjacent to olfr17 DNA in olfr17-expressing cells. **e-f,** Lhx2 and Ebf1 hubs adjacent to olfr17 DNA in olfr17-expressing cells contain old (>24 hr) protein. **g-h,** Lhx2 and Ebf1 hubs adjacent to olfr17 DNA in olfr17-expressing cells contain old (>48 hr) protein. Line scans are marked on figures with dotted white lines and arbitrary intensity units are plotted over distance. All scale bars are 5 μm. **i,** Distance of olfr17 to transcription factor hubs. No significant difference exists between any two measurements. (n = 10 cells, boxes indicate quartiles, center bars indicate medians, red crosses represent outliers, one-way ANOVA with post-hoc Tukey test)

Upon confirming the existence of solid Lhx2/Ebf1 condensates *in vivo*, we asked if they represent transcriptionally engaged hubs or simply structural components of genome folding. To distinguish between these two possibilities, we devised a genetic strategy for the fluorescent labeling of the transcriptionally active Olfr17 allele in our cultured OSNs. Transcription of this OR is not only genetically dependent on continuous Lhx2 expression, but this allele also interacts with a GIH in almost every OSN analyzed by Dip-C. Thus, visualizing the transcriptionally active OR allele offers the ideal system to explore the relationship between Lhx2- and Ebf1-containing condensates, GIHs, and transcription. We used sgRNAs to direct dCas9 to the GFP-tagged Olfr17 allele and incorporated a signal amplification system that would enable visualization of a unique (i.e. non-repetitive) genomic locus. Specifically, we fused dCas9 to a SunTag tail, which is then bound by scFV-mKate2 molecules^56^ (schematic Fig. 5a). We delivered 7sgRNAs, dCAS9, scFV-mKate2 with three different lentiviruses that co-infected the cultured GFP^+^ OSNs, successfully labeling and visualizing the transcriptionally active OR allele in live cells (Fig. 5b).

**Extended Data Fig 6:**
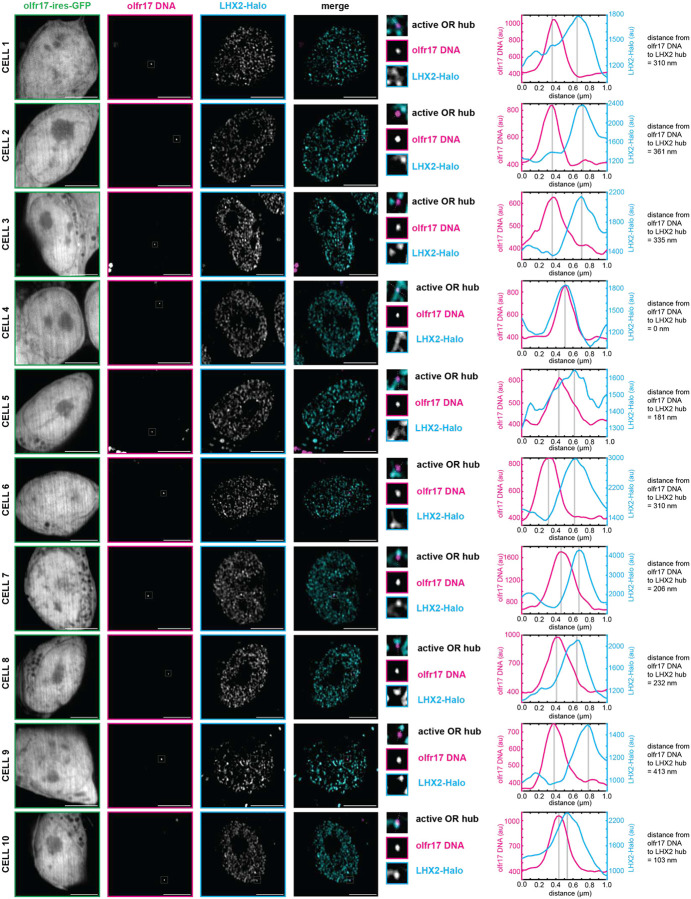
LHX2 Condensates Localize to Active OR Allele *in vivo*. Lhx2 forms hubs adjacent to olfr17 DNA in olfr17-expressing cells. Line scans are marked on figures with dotted white lines and arbitrary intensity units are plotted over distance. All neurons are 6 days old. Scale bars are 5 μm.

**Extended Data Fig 7:**
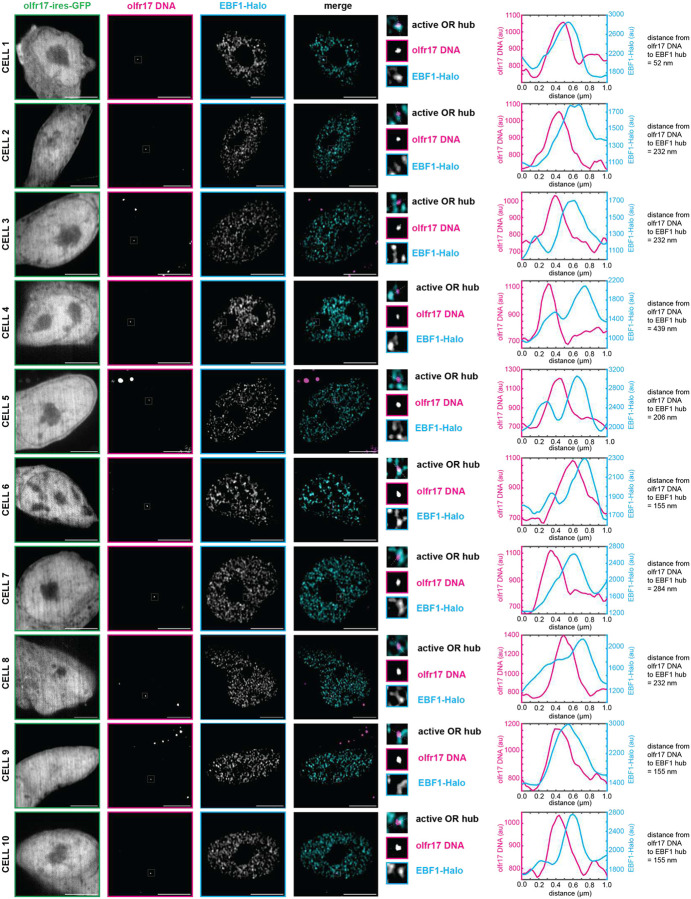
EBF1 Condensates Localize to Active OR Allele *in vivo*. Ebf1 forms hubs adjacent to olfr17 DNA in olfr17-expressing cells. Line scans are marked on figures with dotted white lines and arbitrary intensity units are plotted over distance. All neurons are 6 days old. Scale bars are 5 μm.

**Extended Data Fig 8:**
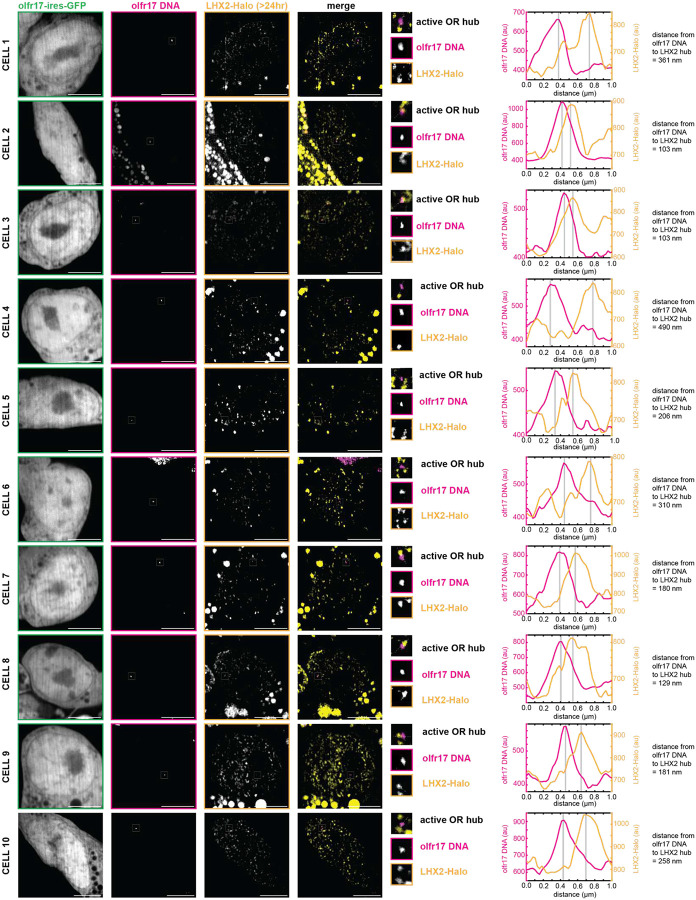
Stable LHX2 Condensates with Protein >24 hr Old Localize to Active OR Allele *in vivo*. Lhx2 protein was stained with JF646 HaloTag Ligand and then incubated with 7BRO for 24 hr to label all old protein with far-red fluorescence. Lhx2 hubs adjacent to olfr17 DNA in olfr17-expressing cells contain old (>24 hr) protein. Line scans are marked on figures with dotted white lines and arbitrary intensity units are plotted over distance. All neurons are 6–7 days old. Scale bars are 5 μm.

**Extended Data Fig 9:**
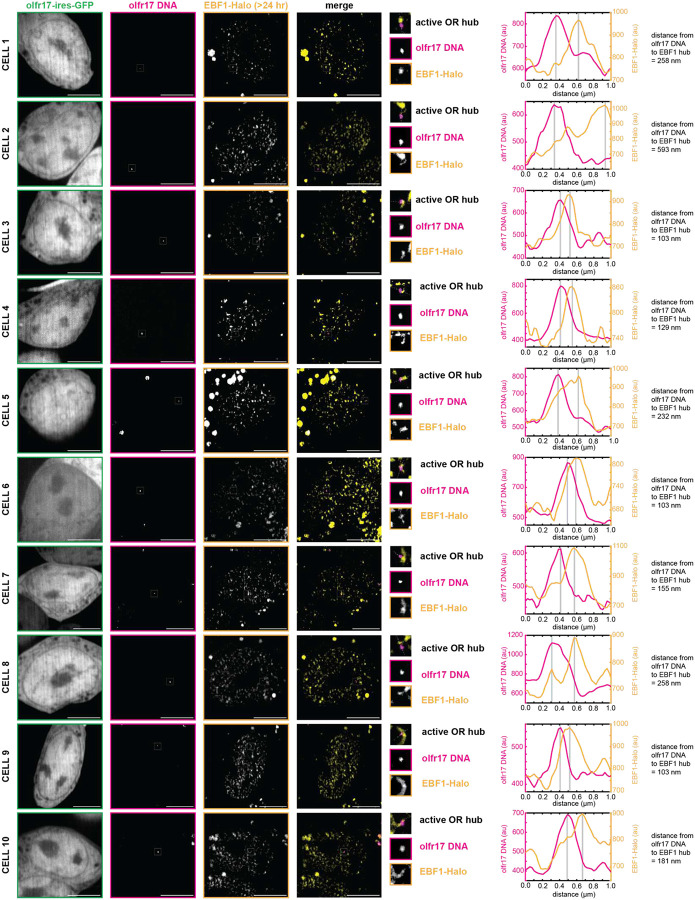
Stable EBF1 Condensates with Protein >24 hr Old Localize to Active OR Allele *in vivo*. Ebf1 protein was stained with JF646 HaloTag Ligand and then incubated with 7BRO for 24 hr to label all old protein with far-red fluorescence. Lhx2 hubs adjacent to olfr17 DNA in olfr17-expressing cells contain old (>24 hr) protein. Line scans are marked on figures with dotted white lines and arbitrary intensity units are plotted over distance. All neurons are 6–7 days old. Scale bars are 5 μm.

**Extended Data Fig 10:**
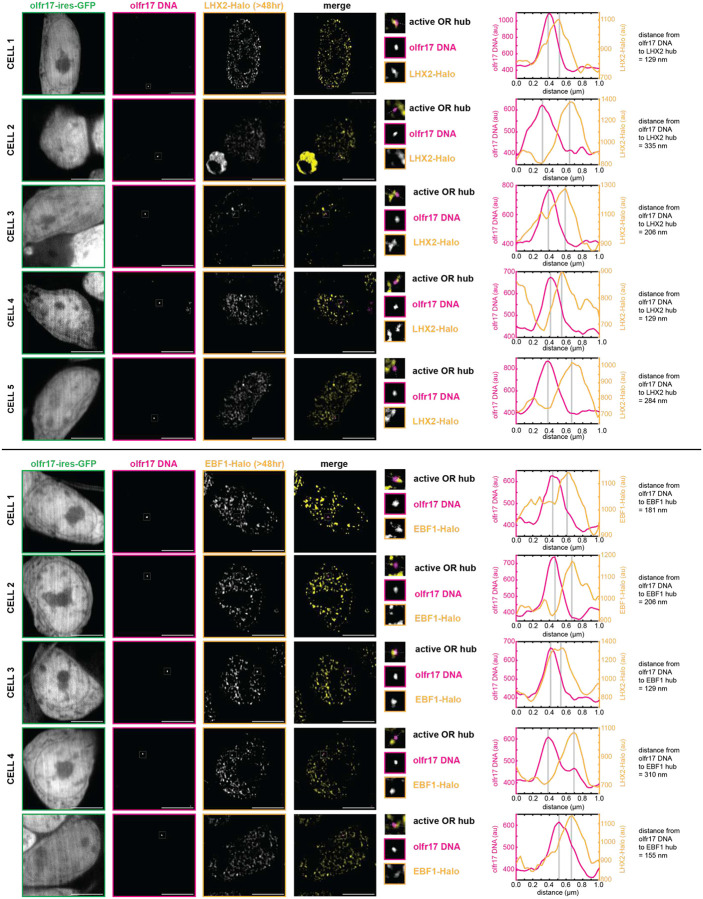
Stable LHX2 and EBF1 Condensates with Protein >48 hr Old Localize to Active OR Allele *in vivo*. Lhx2 and Ebf1 protein were stained with JF646 HaloTag Ligand and then incubated with 7BRO for 48 hr to label all old protein with far-red fluorescence. Transcription factor hubs adjacent to olfr17 DNA in olfr17-expressing cells contain old (>48 hr) protein. Line scans are marked on figures with dotted white lines and arbitrary intensity units are plotted over distance. All neurons are 6–7 days old. Scale bars are 5 μm.

To explore the relationship between the active OR allele and the Lhx2 and Ebf1-containing condensates, we labeled Olfr17 in Halo-Lhx2 and Halo-Ebf1 expressing OSNs. Consistent our hypothesis that solid phase transitions drive assembly of transcriptionally competent GIHs, we observe partial overlap between Olfr17 and Lhx2/Ebf1 condensates in most OSN nuclei (Fig. 5c, d, Extended Data Fig.6–7). In fact, on average Lhx2 condensates reside within 245nm from Olfr17, whereas Ebf1 condensates reside within 214nm from Olfr17 (Fig. 5e). Considering recent reports that super-enhancers can induce transcriptional bursting on the Sox2 locus when these two loci are within 1um from each other^57^, an average distribution <250nm is consistent with functional engagement between Lhx2 and Ebf1 condensates and Olfr17 transcription. Unfortunately, we were not able to perform SMT exclusively at these condensates, due to rapid photobleaching of the fluorescent signal at the active OR allele. Due to lack of available colors, we could not perform pulse/chase experiments with Halo ligands either. However, we were able to label Lhx2 and Ebf1 for 24-hours with a far-red ligand, wash the far-red ligand, and then provide saturating concentrations of a non-fluorescent Halo ligand, assuring that only Lhx2 and Ebf1 proteins expressed during that window are fluorescent. Since we did not observe significant fluctuations in the number of condensates containing only old protein, we imaged cells at 24- and 48-hours after washing the far-red ligand. With this modified pulse/chase protocol we detect numerous OSNs with old Lhx2- and Ebf1-containing condensates at a distance less than 250nm from the active Olfr17 allele, consistent with the solid phase properties described for any randomly selected Lhx2-containing condensate (Fig. 5f–i, Extended Data Fig.8–10). This immediately suggests that solid-like Lhx2/Ebf1 condensates observed throughout the OSN nucleus can be engaged with OR transcription, and likely transcription of other genes.

## Discussion

Our experiments suggest that the DNA sequence alters the homotypic properties of Lhx2/Ebf1/Ldb1 complexes, acting as an instructor of ultra long-range, highly specific, and unusually stable genomic contacts. DNA sequence induced homophilic protein interactions could be caused by the spacing of Lhx2 and Ebf1 sites, as it has been described for the IFNß enhanceosome^48,49^; by direct allosteric effects of motif variations on the structure of the three proteins; or by a combination of the two mechanisms. In either case, our data show that composite motifs of GI enhancers are essential for strong *trans* interactions and solid phase transitions, which could explain both the exclusivity and stability of GIHs. Without a doubt, the composite motif is not the only sequence that can leverage the unique molecular properties of Lhx2/Ebf1/Ldb1, as we detect numerous TF condensates *in vivo*. Indeed, the recent identification of Lhx2/Lhx9/Lef1 motif containing range extending elements (REX) that promote long-range genomic interactions in the mouse limb^58^, together with the demonstration that Ldb1 promotes assembly of widespread but selective multi-enhancer hubs in erythroblast cell lines^59^, suggest that the principles of DNA-induced allostery may not be exclusive to OSNs. However, while protein kinetics may be the same between GIHs and other enhancer hubs, GIs form significantly larger condensates *in vitro* and form stronger *trans* interactions *in vivo*. Most likely, the high concentration of composite motifs in GIs combined with additional unknown sequence features, distinguishes OR enhancers from other CREs, creating a three-tier system in genome organization: GIs with ability to interact with each other across 10s of Mbs and across chromosomes; long-range CREs and REXs that make weaker contacts over long distances; local CREs that are also bound by Lhx2/Ebf1/Ldb1 but lack the sequence features for long-range action. OR promoters, which contain Lhx2 and Ebf1 motifs but lack composite motifs^37,60^, would be assigned to the third tier of regulatory elements, explaining why their recruitment to GIHs requires continuous transcription; if the nascent OR mRNA facilitates recruitment to GIHs^30^, OR transcription would compensate for the significant depletion of composite motifs from OR promoters. Thus, while many GIHs can stably form in an OSN nucleus, transcription-dependent OR recruitment and a recently described RNA-mediated symmetry breaking process^30^, assure that only one OR allele can be stably transcribed in each OSN.

Our discovery raises questions about the value of solid -instead of the widely accepted liquid- phase transitions in genome architecture. Liquid phase separation provides an appealing explanation for the partition of the genome in Mb scale compartments^61^, for the function of super-enhancers^55^, for heterochromatin spreading^44,62^, and for the assembly of the nucleolus^63^. In each of these examples, large genomic segments converge in the nuclear space, exponentially enhancing weak, multivalent interactions through substantial concentration increases of recurrent binders, resulting in liquid phase separation. In the case of GIHs or of the recently described meta-domains^5^, however, the interacting DNA fragments are short (<1Kb) and the total number of converging TF binding sites is small, excluding a sharp concentration increase as the cause of phase separation. Furthermore, weak affinities enhanced by liquid phase transitions, would “trap” Greek Islands locally, in contacts with numerous proximal DNA sequences bound by the same exact proteins. Thus, sequence-induced allostery followed by solid phase transitions may explain why certain DNA sequences “ignore” proximal genomic partners with similar molecular (but not structural) valance and instead assemble specific and stable contacts over vast genomic distances. The unusual stability that solid phase transitions confer to these interactions may represent a genome architecture “engram” in the nucleus of post-mitotic cells. This engram explains the counterintuitive observation that OR and OSN-specific genes regulated by Lhx2/Ebf1/Ldb1 are upregulated in mOSNs, where expression of these proteins is strongly downregulated^30,31^; If slow protein turnover preserves the structural and functional integrity of Lhx2/Ebf1/Ldb1 nucleoprotein condensates, then minimal new protein synthesis would suffice for stable and robust gene expression over the life of the OSN. This novel form of epigenetic memory could be deployed by every post-mitotic neuron that uses Lhx2/Ldb1 complexes for its differentiation^64–67^, as downregulating master regulators without altering nuclear architecture and transcriptional outputs could be crucial for long term maintenance of molecular and cellular identity. Of course, solid nuclear condensates could eventually morph into toxic aggregates, providing possible mechanistic insight to the short OSN lifespan and to the puzzling connection between olfactory deficits and Alzheimer’s disease.

## Methods

### Expression and Purification of Lhx2 Proteins

For truncated protein Lhx21–350 with a stop codon was cloned into a pET28b plasmid (Millipore Cat.69865) so that the final construct 6xHis – Sumo - strep II – Lhx2. A small ubiquitin-like modifier tag (Sumo) was kept on Lhx2 for stability. Full-length (FL) fluorescent Lhx2 protein was also cloned into a pET28 vector so that the final construct contained a 6x His - Lhx2aa1–406 - mKate2 - stop codon.

Truncated Ebf1aa26–422 without a stop codon was cloned into a pET23b plasmid (Millipore, Cat.69746), resulting in a C-terminal His6 tagged Ebf1^1^. Full-length fluorescent Ebf1 was cloned into a pET28 vector. FL Ebf1 was cloned so the final construct contained an mEGFP - Ebf11–591 - 8x His - stop codon.

Ldb1 Lim interaction domain (Ldb1-LID) was cloned into the pET28 vector so that the final construct contained a 6x His - Sumo - Ldb1320–375 - stop codon. FL ldb1 was cloned into a pET-duet vector (MilliporeSigma, Cat.71146) containing a 6x His - Halo tag - Ldb11–411 - stop codon. All proteins were transformed into BL21 E. Coli cells (NEB Cat.C2527I) and grown in LB media. Lhx2 and Ebf1 proteins were grown in media containing 200uM ZnCl2. Lhx2 cultures were grown to OD = 0.6–0.8 and grown at 18C upon induction with 0.3mM IPTG. Ebf1 and Ldb1 proteins were induced with 1mM IPTG. After induction, cultures were grown at 18C for 18–24 hours and then harvested by centrifugation at 4000 rpm for 15 minutes. The pellet was resuspended in a lysis buffer containing EDTA- free Protease Inhibitor Tablet (Pierce Cat.A32965). Lhx2 and Ebf1 protein lysis buffer contained 300 mM NaCl, 2.7 mM KCl, 4.3 mM Na2HPO4, 1.4 mM KH2PO4, 40 mM imidazole, 10% glycerol (v/v), 1 mM TCEP, pH 7.4. Ldb1-LID protein lysis buffer contained 50mM Tris pH 8.0, 400mM NaCl, 10% glycerol (v/v), 10mM imidazole, and 1mM TCEP. FL Halo-Ldb1 protein lysis buffer contained 20mM Tris pH 8.0, 400mM NaCl, 25mM imidazole, 10% glycerol (v/v), 1mM TCEP. All resuspended cells were lysed by sonication and spun at 15,000 rpm for 45 minutes to obtain soluble supernatant.

All supernatants were loaded onto a 5mL HisTrap HP column (Cytiva Life Sciences Cat.17524801), washed, and eluted. Truncated proteins were eluted with linear gradients of elution buffer and FL proteins were eluted with 10 column volumes of elution buffer. Lhx2 and Ebf1 protein elution buffer contained 300 mM NaCl, 2.7 mM KCl, 4.3 mM Na2HPO4, 1.4 mM KH2PO4, 300 mM imidazole, 10% glycerol (v/v), 1 mM TCEP, pH 7.4. Ldb1-LID protein elution buffer contained 50mM Tris pH 8.0, 200mM NaCl, 300mM Imidazole, 10% glycerol (v/v), and 1mM TCEP. FL Halo-Ldb1 protein elution buffer contained 20mM Tris pH 8.0, 400mM NaCl, 250mM Imidazole, 10% glycerol (v/v), and 1mM TCEP. For further Lhx2 and Ebf1 purification, fractions containing protein were pooled, filtered, and immediately loaded onto a 5mL HiTrap Heparin HP column (Cytiva Life Sciences Cat.17040701). The column was washed with buffer containing 20 mM HEPES pH 7.4, 10% glycerol (v/v), and 1 mM TCEP. Protein was eluted with a gradient of 20 mM HEPES pH 7.4, 1.5 M NaCl, 10% glycerol (v/v), and 1 mM TCEP. Fractions containing protein were pooled and analyzed by 4–12% Bis-Tris gels (Invitrogen Cat.NW04125BOX).

After pooling samples, Lhx2 and Ebf1 were dialyzed using 10,000 MWKO SnakeSkin Dialysis Tubing (ThermoFisher Cat.68100). Dialysis buffer for truncated protein contained 20mM HEPES pH 7.4, 200mM NaCl, 10% glycerol (v/v), and 1mM TCEP. Dialysis buffer for FL protein contained 20mM HEPES pH 7.4, 200mM NaCl, 10% glycerol (v/v), and 1mM TCEP. After dialysis, the truncated protein was flash-frozen in aliquots for further use. The full-length protein was concentrated in a 30K Pierce Protein concentrator (ThermoFisher Cat.88531) until the desired concentration is reached. Note that truncated Lhx2 and Ebf1 protein aggregates when being concentrated. To minimize this when a high concentration of protein was needed, we used a 1mL HiTrap Heparin column (Cytiva Life Sciences Cat.17040601) to minimize elution volume, thus concentrating protein. Note that truncated Ebf1 is very unstable and aggregates in cold temperatures and when concentrated. Truncated Ebf1 purification was carried out at room temperature when possible and concentrating this protein was avoided because it caused significant aggregation^1^.

After nickel purification, the elution peak of Ldb1-LID protein was collected and immediately injected onto a HiLoad 26/600 Superdex 75pg size exclusion column (Cytiva Life Sciences, Cat.28989334). Size exclusion was performed in 20mM HEPES, pH 7.4, 200mM NaCl, 10% glycerol (v/v), 1mM TCEP. After size exclusion protein was concentrated in a 3,000 MWKO Pierce Protein concentrator (ThermoFisher, Cat.88525). After concentrating, the protein was frozen in aliquots for further use.

After nickel purification the peak containing FL Halo-Ldb1, sample was dialyzed into 20mM HEPES 7.4, 300mM NaCl, 10% glycerol (v/v), 1mM TCEP. After dialysis sample was incubated with 1:10 molar equivalent of Halo-tag ligand Coumarin dye (Promega, Cat.G8582) for 15 minutes and flash frozen in aliquots. We avoided concentrating FL-Ldb1 when possible. All proteins were run on 4–12% Bis-Tris gels (Invitrogen Cat.NW04125BOX) and analyzed for purity.

### Electromobility Shift Assays (EMSAs)

ATTO-488 labeled forward and unlabeled reverse oligos were ordered from Integrated DNA Technologies (IDT). Probes were mixed in equal-molar rations and then heated to 98C for 5 minutes to fully denature all probes. Next, the probe was cooled to room temperature for 2 hours to re-anneal. Probes were purified using an Oligonucleotide Clean & Concentrator kit (Zymo Research, Cat.D4060).

EMSA reactions contained varying amounts of proteins mixed with 2000 fmol of the labeled probes. For Kd determination, the protein was mixed with DNA probe and then serial diluted to produce titration curves. For standard EMSAs proteins were mixed with master mix and probe and then incubated. All reactions included 1 mg/mL BSA (NEB, Cat.B9200S), 0.1 mg/mL poly Di-DC (Millipore, Cat.118578-37-3), Roche cOmplete protease inhibitors (Millipore, Cat.11836153001), and reaction buffer diluted to a final concentration of 10mM HEPES, 150mM NaCl, 5% glycerol, 0.5% NP-40. Reactions were incubated for 30 minutes at 4C and then run on a 6% native gel using 49:1 acrylamide: Bis acrylamide. Gels were run at 150V in a BioRad Tetrad gel tank and then imaged on a Typhoon FLA 9500 instrument.

### EMSA Binding Curve Analysis

EMSA quantifications were performed using Fiji/ImageJ2. Each lane was quantified by measuring the signal for each band after subtracting background noise. For binding curves, the binding and free probe signal from each lane was converted to fraction bound. Fraction bound was calculated by dividing the signal in each lane by the total signal per lane. The fraction bound was then plotted against the concentration of protein. Each curve was plotted in Prism10 and fit with a nonlinear curve fit. The Kd and hill coefficients were gathered from the curve fit output values. Curves that measured cooperativity assumed a cooperative binding non-linear curve fit. All binding curves were performed in triplicate and plots show standard deviation between replicates.

### Generation of DNA for Condensate Assays

All DNA used in condensate assays was ordered from Twist Biosciences (See table). DNA was then amplified with polymerase chain reaction (PCR) and cleaned then concentrated with Zymo-DNA Clean and Concentrator-25 kits (Zymo, D4033).

### In vitro Condensate Assay

Protein aliquots were thawed immediately before each assay was performed. Full-length proteins were mixed together and diluted to a final concentration of 4uM in 20mM HEPES pH 7.4, 75mM NaCl, and 10% glycerol. DNA was added to a final concentration of 60nM. Components were mixed then transferred to a Superfrost Plus PreCleaned microscope slide (FisherBrand, Cat.12-550-15) with double stick tape to ensure more space between slide and coverslip (Corning, Cat. 2850–22). Slides were imaged after 5 minutes using an Inverted A1R laser scanning confocal microscope. Images were captured with differential interference contrast (DiC) imaging with and without fluorescence. Four images were taken per imaging condition and each condition was imaged in triplicate imaging sessions with independent protein samples.

### Fluorescence Recovery after Photobleaching (FRAP) and Quantification

FRAP was carried out using an AXR MP Confocal microscope. Each channel was bleached with its respective excitation wavelength with a dwell time of 4us and varying power depending on the channel. After bleaching, sample recovery was measured for 10 minutes. FRAP data was quantified using Nikon Software and then normalized by fraction of starting fluorescence. FRAP recovery curves were plotted in Prism10 as the average of triplicate experiments with error bars showing standard deviation between replicates.

### DnaseI Digestion of Condensates

Lhx2, Ebf1, and Ldb1 were mixed together and diluted to a final concentration of 4uM in 20mM HEPES pH 7.4, 75mM NaCl, 10% glycerol, 2.5mM MgCl2, and 0.5mM CaCl2. Lipsi enhancer was added to the reaction to a final concentration of 60nM. 10uL of reaction was incubated for 10 minutes in an Ibidi u-Slide 15 well 3D glass bottom plate (Ibidi, Cat.81507). After incubation 30U of DnaseI (Ambion, Cat.4393898) was gently dripped down the side of the well and into the reaction. Images were collected every 5 minutes for 20 minutes after DnaseI addition. For negative control, an equal volume of sample buffer was added in substitute for DnaseI.

### Quantification of Condensates

DiC images were quantified using Fiji/ImageJ. Our macro used the Canny Edge Detector plugin to identify structures and then fill them in with the gray morphology close, open, and erode functions (respectively). We then quantified the size of all particles and calculated the average condensate size across imaging triplicates. For DiC quantification we plot the average condensate size with error bars showing standard deviation.

Fluorescent images were quantified using Fiji/ImageJ. Our macro subtracted background from all images, set thresholds across all images, and then analyzed particles for each fluorescence channel. For Fig. 1D we quantified the size of all condensates present across images for Lhx2 and Ebf1. We then classified each condensate as being <1um2, 1–5um2, 5–25um2, or >25um2. We calculated the fraction that each class occupied and plotted this distribution in various conditions.

DiC quantification of the DnaseI digestion was carried out Fiji/ImageJ. We calculated the starting condensate size for 10 structures and measured the size of the same condensates after 5, and 10 minutes of DnaseI digestion. We then calculated the fraction of the starting size for each time point and plotted it in Prism10.

### Mice

Mice were treated in compliance with the rules and regulations of the Institutional Animal Care and Use Committee of Columbia University under protocol number AABG6553. Neonatal pups were euthanized via decapitation. Both male and female mice were used for experiments. All live cell experiments were performed on dissociated cells prepared from whole olfactory epithelium tissue. This study used primary cells expressing *olfr17* by crossing *tetO-P2-IRES-GFP* mice to *Gng8(gg8)-tTA* mice^2^. *Ebf1-* and *Lhx2-HaloTag* CRISPR knock-in mice were heterozygous unless otherwise described.

### mOSN Culture

mOSN culture protocol was modified from Gong, 2013^3^. Neonatal pups (P<5) were decapitated, MOE was dissected and isolated into PBS. They were then dissociated with papain for 40 minutes according to the Worthington Papain Dissociation System. Following incubation, the tissue was titurated and filtered through a 40 μm filter and pelleted. Cells were resuspended in mOSN medium (Waymouth’s MB 752/1 medium (Gibco, 11220035), 1% N2 supplement (100x, Gibco, 17502048), 1% Antibiotic-Antimycotic (100x, Gibco, 15240062)). 6 × 10^5^ cells were plated per 35 mm^2^ dish on top of a confluent layer of astrocytes. Any lentivirus was introduced at the same time as plating of neurons. OSN cultures were maintained at 37°C with 5% CO_2_ in mOSN media. 24 hrs after plating, 50% of the mOSN medium was replaced with mOSN medium and afterwards, half of the medium was replaced every other day.

### Astrocyte Culture

Astrocytes were harvested and cultured as previously described^4^. For imaging experiments, primary astrocyte cultures were plated on 35 mm^2^ Collagen Coated Mattek dishes (P35GCOL-1.5–10-C) and maintained at 37°C with 5% CO_2_.

### Lentivirus Production

HaloTag-BRD4 was cloned from (Addgene:183939) into a pFUW vector (Addgene: 14882). The red calcium indicator, jRGECO1a (Addgene: 61563), was cloned into pFUW. DNA labeling constructs and vectors are described in the imaging section below. Lentivirus was produced in the Lenti-X^™^ 293T Cell Line (Takara). Cells were transfected with Lipofectamine3000 (Invitrogen, L3000001) according to the manufacturer’s instructions. To produce virus, the DNA mix contained the gene of interest, the packaging plasmid PAX2 (Addgene: 35002), the envelope plasmid MD2.G (Addgene: 12259), and the rsvREV plasmid (Addgene: 12253). Media was collected 24hr and 72hr post-transfection and pooled. To concentrate the virus, virus-containing media was filtered through a 0.45 μm filter and ultracentrifuged (70,000xg, 2hr, 17°C) on a 20% sucrose cushion. The viral pellet was resuspended in PBS, aliquoted and stored at −80°C.

### mOSN Culture Validation

#### RNASeq:

RNA was extracted from sorted cells using Direct-zol RNA kits from Zymo Research. 100 ng of total RNA was used to prepare DNA libraries with NEBNext single cell/low input RNA library prep kit followed by 61 HO paired-end and multiplexed sequencing. Reads were aligned to mouse genome (mm10) using Subread and the raw read counts were assembled using featureCounts pipeline. Deseq2 was used to detect differences between cultured cells and from the mice’s biological replicates. For cultured neurons we pulled 3 wells per sample and used three replicates. For control we used sorted cells from 3 male mice 5–6 weeks old.

#### Immunofluorescence:

Cells from a 6-day old culture were washed with 1xPBS, fixed with 4% paraformaldehyde (Electron Microscopy Sciences) for 10 minutes at room temperature and washed prior to immunostaining. After fixation, cells were incubated in blocking buffer: 2.5% donkey serum, 2.5% goat serum (Jackson ImmunoResearch Laboratories), 1% bovine serum albumin (Sigma-Aldrich), and 0.1% Triton X-100 (Sigma-Aldrich) for 1–2 h at room temperature. The following primary antibodies were used at the indicated concentration in blocking buffer overnight at 4°C: mouse monoclonal anti-Tubulinβ3 1:500 (TUBB3, 801213, BioLegend), rabbit polyclonal anti-AC3 1:100 (sc-588, C-20, SantaCruz), and rabbit monoclonal anti-NCAM 1:250 (AB5032, Chemicon, Millipore-Sigma). Secondary antibodies against mouse IgG and rabbit IgG conjugated to Alexa Fluor 488 or 594 (Molecular Probes–Thermo Fisher Scientific) were used at a 1:2 000 dilution for 1–2 h at room temperature. DAPI (Sigma-Aldrich) was used at 0.1 ug/mL in PBS.

#### Calcium Imaging:

Cells were infected with a lentivirus containing a red-calcium sensor, jRGECO1a (Dani, 2016). Trans-2-undecenal (W342300, Sigma-Aldrich), an olfr17 ligand (Sandeep Datta, personal communication), was provided 50:50 in mineral oil, replacing 10% of media at time 0 s.

#### DNA FISH:

Experiment was performed as described previously using the panOR probe^5^.

#### HiC:

We used 10–30 thousand cells for Hi-C. Sorted cells were lysed and processed through Hi-C protocol as previously described^6^ . In brief, cells were lysed with 10 mM Tris pH 8 0.2% Igepal, 10 mM NaCl. Pelleted intact nuclei were then resuspended in 0.5% SDS and incubated for 20 min at 62 °C for nuclear permeabilization. After being quenched with 1.1% Triton-X for 10 min at 37 °C, nuclei were digested with 25 U/μl MseI in 1× CutSmart buffer for 1.5 hours at 37 °C. Following digestion, the restriction enzyme was inactivated at 62 °C for 20 min. For the 45-min fill-in at 37 °C, biotinylated dUTP was used instead of dATP to increase ligation efficiency. Ligation was performed at 25 °C for 30 min with rotation after which nuclei were centrifuges. To degrade proteins and revers crosslinks pellets were incubated overnight at 75 °C with proteinase K. Each sample was transferred to Pre-Slit Snap-Cap glass mictoTUBE and sonicated on a Covaris S220 for 90 sec. Sonicated DNA was purified with 2× Ampure beads following the standard protocol and eluted in 300 μl water. Biotinylated fragments were enriched as previously described using Dynabeads MyOne Strepavidin T1 beads. The biotinylated DNA fragments were prepared for next-generation sequencing directly on the beads by using the Nugen Ovation Ultralow kit protocol. DNA was amplified by 8 cycles of PCR. Beads were reclaimed and amplified unbiotinylated DNA fragments were purified with 1× Ampure beads. The quality and concentration of libraries were assessed using Agilent Bioanalyzer and Qubit Quantification Kit. Hi-C libraries were sequenced paired-end on NextSeq2000 (2 × 65 bp). Raw fastq files were processed using the Juicer single CPU BETA version on AWS. After reads are aligned, merged, and sorted, chimaeras are de-duplicated and finally Hi-C contact matrices are generated by binning at various resolutions and matrix balancing. In this paper we present data with stringent cutoff of MAPQ >30. Hi-C matrices used in this paper were matrix-balanced using Juicer’s built-in Knight-Ruiz (KR) algorithm. Matrices were visualized using Juicebox.

#### B- Cell Validation:

Spleens from 6 week old mice were harvested directly into a 40um filter holding 2mL ACK lysis buffer (homemade) and smashed with the back of a syringe. They were incubated for 4 min at RT and then quenched with 10 mL 1xPBS. Cells were spun at 1500 rpm for 5 min at 4C. Supernatant was removed and cells were resuspended in FACs buffer (0.5% BSA, 5mM EDTA, 15mM HEPES in Ca++/Mg++ Free PBS). Cells were resuspended in 1:200 B220-FITC (Biolegend, 103205) and 1:100 Fc Block (BD Biosciences, 553141) diluted in FACs buffer. Cells were incubated for 20 min at room temperature in the dark. Cells were washed in double the volume used for staining then spun at 1500 rpm for 5 min at 4C. Cells were then resuspended in 200 uL FACS buffer with 1:100 DAPI and run on a Sony MA900. 200k cells were recorded per condition and analyzed with FlowJo.

**Cut&Tag (Vazyme, #TD904)** was performed according to the manufacturer’s instructions to identify Lhx2 binding sites in olfr17-expressing neurons. Three biological replicates were conducted. Sequencing was performed using the Illumina P2–100 kit on a NexSeq 2000 platform. Raw reads were mapped to the mouse genome mm10 using **Bowtie2**^7^. Sam files were converted to bam files and sorted by Samtools^8^. **MACS2** was employed to call binding peaks^9^. The final Lhx2 binding peaks were identified by retaining only those peaks that were consistently detected across all three biological replicates.

To identify composite motifs, **EMBOSS Fuzznuc**^10^ was used to search for the following Lhx2-Ebf composite motif pattern across both strands of the mouse genome: (TA)(TA)A(TCA)(TGA)(AG)(AG)(CGT)(CTA)(CTA)(CTA)(CTA)N(GAT)(GA)(GAT)(GAT)[4]. Lhx2 binding peaks containing this composite motif were identified using the bedtools intersect function, with the setting to intersect Lhx2 binding peaks that contained at least 90% of the sequence of the composite motif^11^.

### Circos Analysis:

Hi-C data for P2 neurons were generated by merging biological replicates from the olfr17-ires-GFP and TetO-olfr17-ires-GFP mouse lines from previous publications^12,13^. Hi-C raw data for mOSNs were downloaded from the 4DN Nucleosome database (Data file ID: 4DNFI1MX8L3L).

The **HiCPro** pipeline^14^ was used to process and analyze the Hi-C data, generating ICE-normalized contact matrices at 5kb resolution for olfr17 neurons.Trans contacts within Greek islands, as well as those within Lhx2 binding sites containing the composite motif, were extracted from the ICE-normalized contact matrix at 5kb resolution for olfr17-expressing neurons. A cutoff value of 2 for ICE-normalized contact probability was applied to subset strong trans contacts. Circos plots of strong trans contacts were generated using the **circlize** package in R^15^. Trans contacts within 9 Greek islands (GI) and 9 non-GI Lhx2-Ebf co-bound regions were extracted from the ICE-normalized contact matrix at 20kb resolution for mOSN.

### Imaging

#### Olfr17 dCas9 DNA Label:

7 top guides were designed using the Benchling (benchling.com) CRISPR Tool. A modified dCas9 binding hairpin used as previously described to enhance binding^16^. The designed sgRNA cassette was synthesized via Genscript and then cloned into a pLVX lentivirus vector (Takara). A dCas9-SunTag^17^ (24 copies of a GCN4 peptide fused to the C-terminus of dCas9, Addgene: 60910^17^) was cloned into a pFUW vector (Addgene: 14882). In order to fluorescently label the SunTag in red, scFv-GCN4-sfGFP-GB1 (Addgene: 60906) protein was modified by replacing sfGFP with mKate2 (Addgene: 104009), and the entire protein was then cloned into the pLVX vector. All three components of the labeling system were packaged into lentiviruses and introduced when plating the neurons.

#### HaloTag Staining:

TMR-Direct HaloTag Ligand (G2991, Promega) was supplied in media at 1:1000 per manufacturer’s protocol for at least 24 hours. JF-646 HaloTag Ligand was supplied in media at 1:500 for standard imaging and 1:4 000 000 for single molecule imaging. 7-bromoheptanol (7BRO, H5476203, ThermoScientific) was used as a HaloTag blocking agent at 10 um, as described^18^. For pulse-chase experiments, cells were incubated with media containing TMR-Direct HaloTag Ligand or JF-646 HaloTag Ligand for at least 24 hr and then washed with media 3x and then incubated with media containing JF-646 HaloTag Ligand or 7BRO for the duration of the “chase.”

#### Microscopy:

Single molecule imaging was performed on a Bruker Vutara VXL. All other OSN imaging was performed using a CSU-W1 SoRa Yokogawa spinning disk confocal microscope with Optical Re-Assignment. Both microscopes were located at the Zuckerman Institute Imaging Platform. SoRa images were deconvolved with Microvolution software (https://www.microvolution.com/). Single molecule localizations were performed with Bruker Vutara software, tracking was performed using simpletracker, with a maximum linking distanceof 400 nm and no gaps allowed (Jean-Yves Tinevez, https://github.com/tinevez/simpletracker). Localizations were defined as being inside a condensate if >4 localizations were found within 7.5 nm during the total imaging time. Pulse chase analysis was performed using an author-written analysis function written in MATLAB (Supplemental Information).

## Extended Data
